# Integrated pest management in the academic small greenhouse setting: A case study using *Solanum* spp. (Solanaceae)

**DOI:** 10.1002/aps3.11281

**Published:** 2019-08-09

**Authors:** Daniel S. Hayes, Ingrid E. Jordon‐Thaden, Jason T. Cantley, Angela J. McDonnell, Christopher T. Martine

**Affiliations:** ^1^ Biology Department Bucknell University 1 Dent Drive Lewisburg Pennsylvania 17837 USA; ^2^ Department of Botany University of Wisconsin 430 Lincoln Drive Madison Wisconsin 53706 USA; ^3^ Department of Biology San Francisco State University 1600 Holloway Avenue San Francisco California 94132 USA

**Keywords:** agriculture, cultivation, education, nightshades, plant pests, plant–insect interactions, *Solanum*

## Abstract

**Premise:**

Botanical faculty and staff at academic institutions are often tasked with establishing and/or caring for plant collections held in small greenhouse facilities. Once plants are in place, an especially acute challenge is managing plant pest/pathogen populations. Integrated pest management (IPM) approaches are an excellent option, but few examples exist in the literature of successful programs that have been developed in academic small greenhouse settings.

**Methods and Results:**

Over several years, we developed an IPM program for two small research greenhouses on the campus of a primarily undergraduate institution where hundreds of plants have been grown for studies in the genus *Solanum*. We here present a synopsis of the cultural, mechanical, physical, and biological controls used as part of our successful IPM strategy—including details on the efficacy of multiple predatory insects—with the hope of providing a model for sustainable pest management in the higher education environment.

**Conclusions:**

IPM can be an effective strategy for maintaining healthy plant populations in small research greenhouses, but it requires a consistent investment of time and funding. A well‐cared‐for plant collection might help support numerous positive outcomes, including advances in faculty scholarship and opportunities for student learning and/or training.

Small greenhouses are important resources for many academics working in the plant sciences. These spaces, when available and operative, are especially integral to research and teaching in the higher education context—where their care and maintenance is often left to an individual faculty member. Management of these spaces can be time consuming and challenging, but is particularly demanding in terms of plant care requirements—most notably in the area of plant pest management, where the use of insecticides and other treatments may require training, licensing, and risks to applicator health. In an attempt to address these issues and create student learning opportunities, we established an integrated pest management (IPM) scheme in our research greenhouse space at Bucknell University, a small, private, liberal arts institution focused on undergraduate education. It is our opinion that more plant scientists at primarily undergraduate institutions should consider IPM for their living collections, and we believe that the following assessment of what we have learned over the past seven years can be of use in that regard.

Novel integrated biological and chemical control studies by California entomologists formed the basis of what we now call integrated pest management, or IPM: an ecosystem‐based strategy focused on the long‐term prevention of pests through a combination of practices and techniques that minimize risks to human health and the environment (Smith and Smith, 1949; Michelbacher and Bacon, 1952; Stern et al., 1959; Kogan, 1998). Integrated control and pest management gradually became synonymous under the concept of IPM, although over the past 50 years the definition of IPM has become more pliable as it has been applied to fit governmental and local interpretations (Van Lenteren and Woets, 1988; Bajwa and Kogan, 2002; Ehler, 2006; Barzman et al., 2015). Despite relative growth of IPM in the 1990s, optimistic researchers grew frustrated with the lack of widespread adoption, citing the lack of outreach and knowledge‐sharing as fundamental to slow implementation (Kogan, 1998; Ehler and Bottrell, 2000; Van Lenteren, 2000, 2012; Ehler, 2006). More recently, promoters of IPM have called for an increase in IPM extension, publications, and knowledge, citing a general dearth of experienced users, knowledgeable consultants, and IPM initiations (Van Lenteren, 2012; Parsa et al., 2014; Barzman et al., 2015; Buurma and van der Velden, 2017; Parrella and Lewis, 2017; Lamichhane et al., 2018). Although a call for increased IPM education has long been made, many researchers and practitioners have recognized a need for increased flexibility in IPM programs as well as better knowledge‐sharing among users with locally adapted programs (Barzman et al., 2015; Benjamin and Wesseler, 2016; Buurma and van der Velden, 2017; Giles et al., 2017; Lamichhane et al., 2018).

A thorough review of IPM literature yields few peer‐reviewed journal publications describing IPM programs for small research greenhouses, and no record of implementation for the economically and biologically important genus *Solanum* L. As part of our work on the evolution and ecology of reproductive systems in *Solanum* taxa, we have cultivated at any given time over the course of the past seven years between two and 25 individuals of up to 30 different taxa (sometimes growing hundreds of plants, sometimes only a small number). Although many plants in the genus, like other members of the nightshade family (Solanaceae), exhibit herbivore defense via secondary metabolites (and other strategies), greenhouse‐grown solanums are notorious for developing aphid, whitefly, thrips, and spider mite populations in the absence of the natural enemies that normally suppress them in outdoor fields (Carol Glenister, IPM Labs, personal communication). In response, researchers growing non‐crop species indoors often resort to the use of chemical applications. Meanwhile, in parallel, facilities for growing crop species (like tomatoes) have universally adopted IPM in large part because bumblebees are required for pollination and fruit set (Carol Glenister, IPM Labs, personal communication).

In this paper, we review the process of developing a successful IPM strategy for small research collections. Ongoing work in our lab requires the growth and maintenance of multiple Australian Monsoon Tropics (AMT) *Solanum* species, often for months or years at a time, in a relatively small (10 × 12 m) rooftop greenhouse and an accompanying ground‐level greenhouse of similar size. Recognizing that IPM is shaped according to site‐specific and taxon‐specific factors, we provide arguments and examples of how dynamic and flexible IPM strategies can be translated into modern research greenhouses. We provide an example of how the substantial existing knowledge on pests, parasites, and predator–prey relationships can be combined with practical experience into a working system of guidelines that is usable by academic plant scientists to develop their own IPM system—whether they work on *Solanum* or any other plant taxa.

## METHODS AND RESULTS

In this section, we present a case study of our experience developing an IPM program and examine the merits of an IPM strategy heavily reliant on beneficial insects. The following segments will cover the establishment of a protocol centered on pest‐specific thresholds, creation of an IPM seasonal calendar, and implementation of scouting guidelines and checklists. This is not an encyclopedic review of IPM nor a one‐size‐fits‐all prescription for pest problems, but, rather, one example of a successfully established program. Although there exists an abundance of knowledge on greenhouse predator–prey relationships and seasonal beneficial insect ordering recommendations, nearly all such material is produced by IPM suppliers or large state‐funded agricultural programs. As such, starting an IPM system is often challenging and intimidating for small research programs and new greenhouse managers tasked with establishing pest control in the academic setting. Considerations related to specific growing goals (e.g., how many plants will be grown and for how long) and resource commitment (time, effort, funding) vary from institution to institution.

### Cultural controls

In a small growing space supporting a multi‐species plant community, fostering a beneficial insect community requires early establishment of basic cultural controls with the aim of maintaining healthy plant growth while complementing natural plant resistance. Institutions utilizing IPM may need to support a heterogeneous research agenda in which the development of diverse research projects can greatly influence what taxa are grown, how many individuals are in culture, and how long plants need to be maintained. To address some of the challenges of a research schedule that is inherently tied to an academic calendar, a rough identification system was assigned to divide plants into germination (G), vegetative growth (V), and reproductive (R) stages. Use of this identification system allows for temporal and spatial segregation of plants in use across multiple small research projects, particularly as student availability or involvement ebbs and flows. For plants cultured over long time periods, these phases may overlap as reproductive stages can extend for months or years.

In our collection, most individuals are germinated (G) during the winter months and are maintained for two to three months or longer post‐germination (V) before being capable of reproduction (R). Although blooming flowers and large inflorescences present a new opportunity for pests to establish, preventing pest infestation during G and V stages is weighted higher. Pest outbreaks during G and V stages are often easier to identify and combat, but also often retard subsequent floral development. To promote plant health and reduce pest susceptibility during G and V stages, plants are isolated, as best as possible, from older R plants. Growth chambers, when accessible, are a convenient means to bolster G and V growth in an isolated pest‐free environment. Moving plants to secluded corners under growth lights has also been used to hinder the spread of pests from established R populations by establishing a physical distance between populations while still keeping the plants growing in the greenhouse. To bolster natural plant resistance and promote continuous growth, fertilizer was applied during repotting and, as needed, via slow‐release pelleted fertilizer and foliar sprays (see Appendix Overview of common nutrient deficiencies encountered during *Solanum* spp. growth. Healthy plants are less susceptible to pests and thus require less IPM investment. for notes on common nutrient deficiencies and fertilizer treatments). To establish a healthy beneficial insect community while promoting plant health, beneficial insects in early development stages are applied directly to soil during repotting.

Most of the beneficial insects that our program has used achieve optimal predation in temperatures of 19–27°C and thrive in higher relative humidity (Table 1). Beneficial insects for growing conditions outside of these ranges can be identified and purchased through one's biocontrol provider. The set of *Solanum* species that we maintain are adapted to warmer, semi‐arid conditions of northern Australia (e.g., Kakadu National Park, 17–30°C night to day). Temperature controls for the greenhouse are set to 25°C, night and day, to encourage sustained plant growth while accommodating ideal temperatures for maximizing beneficial predation of pests. Our main greenhouse is of glass construction and is situated on the rooftop of the biology building with cement floors and building water. Our second greenhouse is a poly hoop house with gravel floors. Both greenhouses are used year‐round. The artificial lighting (high‐pressure sodium), exhaust fans, and temperature are controlled automatically by Wadsworth Control Systems (Arvada, Colorado, USA). Our glass greenhouse did not have additional humidity added, hovered between 40–70% seasonally, and was lower in humidity than our hoop house, given its isolation on top of the biology building. Other adjustments to the climate included the seasonal use of artificial lighting, two adjustable‐direction ceiling fans in the greenhouse, horizontal fans, steam‐fed heaters, evaporative cooling, exhaust fans, and movable floor fans set on simple timers. Air flow was kept to a maximum to help reduce hot spots for insects and mold.

**Table 1:** Overview of beneficial insect lifecycles

Organism	Optimal environment^a^	Lifespan	Target spp. stage	Beneficial predatory stage	Release stage	Release rate used
*Aphidoletes aphidimyza*	65–77°F, 70–90% RH	4 wk immature stages, 2 wk adult	Adult	Larval	Adult, shipped as pupae	0.5–1 per 5–10 sq ft
*Coccinella septempunctata*	61–82°F	1 wk egg, 2–3 wk larva, 1 wk pupa, 2–3 mo adult	Adult	Larval–adult	Adult	1 per sq. ft.
*Chrysoperla rufilabris*	60–90°F	2–6 d egg, 2–3 wk larva, 1 wk pupa, 1 wk adult	Adult	Larval	Egg, available as larvae too	1–3 per sq ft (prevent)
*Aphidius colemani*	60–80°F, 80% RH	2 wk immature, 2 wk adult	Nymph–adult	Egg–adult: parasitoid	Adult, shipped as mummies	5.5 per sq ft‐outbreak
*Aphidius matricariae*	60–75°F, 60–80% RH	10 d immature, 2 wk adult	Nymph–adult	Egg–adult: parasitoid	Adult, shipped as mummies	1–2 per sq ft (low qt. pest)
*Hypoaspis miles*	Soil mite	1–2 d egg, 5–6 d nymph, 7–11 d adult	Larval	Adult	Adult	1 L per 1000 sq ft
Scanmask *S. feltiae*	Soil nematode	18 d	Larval	Adult	Nematodes	1 L per 1000 sq ft
*Amblyseius cucumeris*	70°F, 70% RH	3 d egg, 2 d larva, 7 d nymph, 1 mo adult	Egg	Pupa–adult	Egg	1 sachet per 10 sq ft
*Orius insidiosus*	70–90°F, 60% RH	4–5 d egg, 2–3 wk nymph, 3–4 wk adult	Larval–adult	Adult	Adult	1 per 10 sq ft
*Encarsia formosa*	60–80°F, 70% RH	3 wk immature stages, 1 mo adult	Immature stages (2–4)	Egg–adult: parasitoid	Egg	1 per 3–6 sq ft
*Cryptolaemus montrouzieri*	75–80°F, 60% RH	3 wk immature stages, 1 mo adult	Larval–adult	Adult	Adult	2–5 per sq ft
*Phytoseiulus persimilis*	65–80°F, 60% RH	8 d immature stages, 1–2 mo adult	Egg–adult	Adult	Egg sachets	0.5–2 per sq ft
*Mesoseiulus longipes*	70–90°F, 40% RH	8 d immature stages, 1–2 mo adult	Egg–adult	Adult	Adult	1 per 3–5 sq ft
*Neoseiulus californicus*	50–110°F, 50–60% RH	8 d immature stages, 3 wk adult	Egg–adult	Adult	Adult	0.5–2 per sq ft

Note

RH = relative humidity.

a Optimal conditions for performance of the beneficial insect, not prerequisites for successful implementation.

### Mechanical and physical control

Because our smaller greenhouse environment has limited space for separations and barriers, mechanical and physical controls are often not implemented. General greenhouse cleanliness is thus an important tool for eliminating reproductive hot spots for pests. Greenhouse floors are washed with water weekly, and drains and grates are cleaned monthly. Dying and dropped leaves are removed daily prior to watering. Leaves infested with pests prior to release of beneficial insects are removed to hinder spread. Some pests, such as aphids, mealybugs, and flea beetles are also hand squashed and removed manually from foliar surfaces. A small amount of time investment day‐to‐day can save hours and dollars down the road fighting an uphill battle against established pests on neglected plants.

Appropriate watering techniques are paramount in aiding and controlling biological controls and pests. The growth and spread of powdery mildew fungi (Erysiphales) on leaves was often a significant problem early in IPM establishment, severely stunting plant growth. Switching to low‐spray hose nozzles that limit the water mist created during application was an important step in solving much of this problem, as was moving from gravel‐filled bench tops to benches topped with a drainable grid. During watering, hose nozzles should not touch surrounding soil and leaves in an attempt to limit the spread of pest species with soil‐dwelling stages of their life cycles. Beneficial insects (e.g., *Aphidoletes cucumeris* Lint) also contain soil‐dwelling stages in their life cycles and overwatering can dispel soil and larvae from pots.

Even in our small space, open pathways between plant benches aided in creating artificial barriers some pests had trouble crossing. Plants are often grouped by species, age, and/or size, creating additional vegetative barriers to pest movement. With small physical barriers and limited transportation between benches, we saw pest outbreaks contained to respective benches and plant groups. Pests do not often migrate between benches or plant species, aiding in control and management. As with many aspects of IPM, this approach took time to discover and trial and error to implement effectively.

### Biological control

Heavy emphasis was placed on biological control as the main focus of our IPM system. In contrast to what is often described as IPM, but perhaps better identified as integrated pesticide management (Ehler, 2006), we used chemical controls as a last‐resort measure for recalcitrant pests, relying on natural predators and parasites for prevention and outbreak control.

Daily scouting of all individual plants was critical to early detection of pests. Virgin symptoms were thoroughly researched, using Malais and Ravensberg (2003) as an initial reference. Biological treatments were designed in correspondence with IPM Laboratories (Locke, New York, USA) and BioLogic Company (Willow Hill, Pennsylvania, USA). Timelines for seasonal pest establishment were noted, and various biological treatments tested for efficacy and longevity. Yellow sticky traps were particularly useful in identification of truculent pests often not visible on plant foliage to the naked eye, although these can also trap beneficial insects and should only be used over a 36‐h period to assess current pest loads.

### Observations and results of biological treatments for common greenhouse pests

#### Aphids (Aphididae)

Aphididae species were often associated with seasonal shifts. For instance, *Myzus persicae* (Sulzer), the green peach aphid, was especially prone to outbreaks during spring and fall months. Aphids are particularly detrimental to plant hosts due to their specialized feeding behavior (see Miles 1999 for analysis of phloem sap extraction and Brault et al. 2010 for plant virus transportation). Aphid outbreaks occurred multiple times during the reported timeframe, with infestations most commonly happening during the transitional weeks between fall–winter and winter–spring. As with many pests, we could not be certain if aphids colonized the greenhouse independently on numerous occasions or if low baseline levels of aphids (typically depressed by predatory insects) expanded quickly enough to temporarily overwhelm our IPM during those periods.

**Figure 1:**
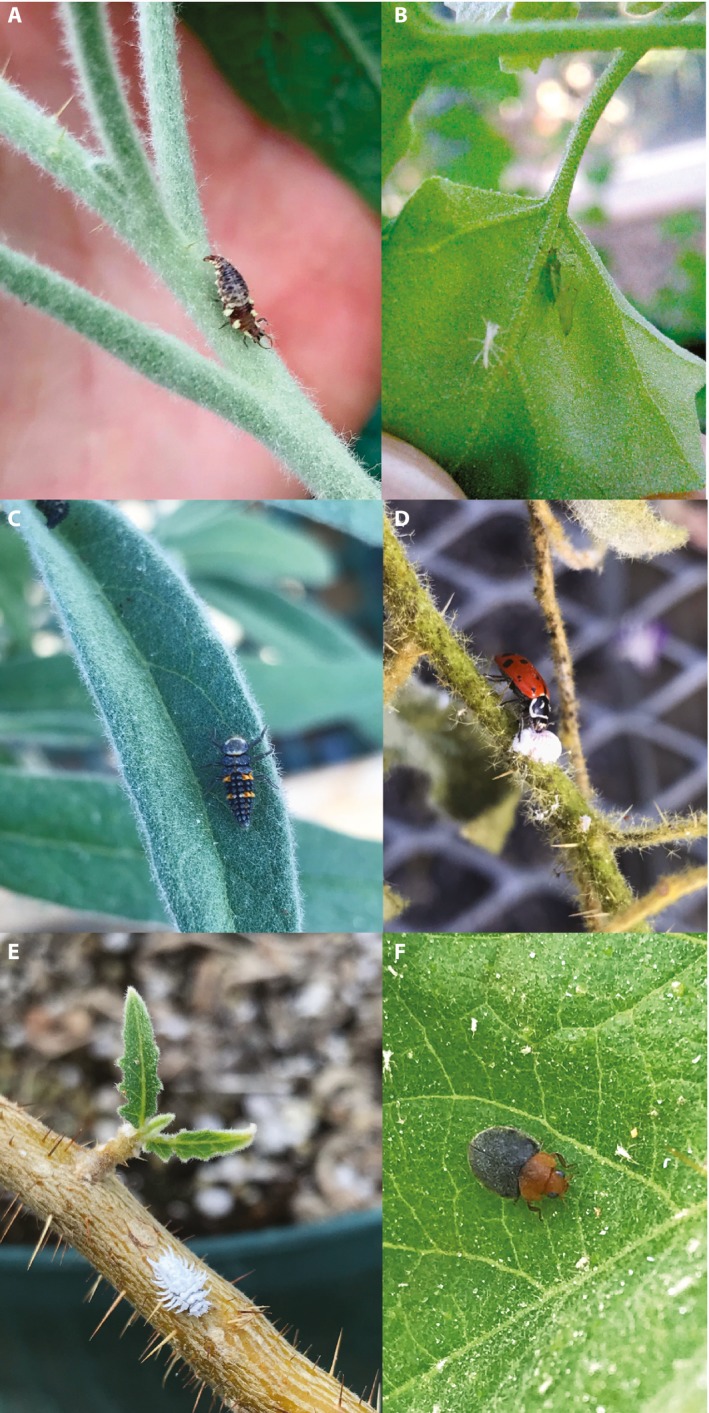
Three of the most common predatory insects in our standing IPM order used to help control aphids and mealybugs, two of the primary pests of greenhouse‐grown *Solanum*. (A, B) Green lacewing (*Chrysoperla rufilabris*) larval and adult stage (adult recently emerged). (C, D) Ladybird beetle (*Hippodamia convergens*) larval and adult stage (adult preying on mealybug). (E, F) *Cryptolaemus montrouzieri* larval and adult stage. All photos by A.J.M. except B by S. Long.

The common green lacewing, *Chrysoperla rufilabris* (Burmeister) (Fig. 1A, B), was a preventive beneficial insect employed to naturally control aphid establishment and was primarily introduced through slow‐release hanging sachets (pouch), where insects would grow from egg stages before emerging as larvae/adults (Tables 1, 2). Although adults have been known to survive and actively reproduce during their 4–6‐wk lifespans (Legaspi et al., 1994), no adults have been observed actively reproducing in our space. *Chrysoperla rufilabris* served as an effective preventive measure for aphid outbreaks, successfully preventing establishment outside of early spring periods.

**Table 2:** Synopsis of common insect pests/pathogens encountered during period of study and biological control employed.

Pest (family/species name)	Beneficial organism release (species name)	Quantity (for 100–400 plants)	Suggested schedule	Notes on efficacy
Aphid (Aphididae)	Gall midge (*Aphidoletes aphidimyza*)	200 adults	p.r.n. for outbreaks: biweekly (2 wk) to monthly (mo)	Highly effective, quick acting, kills more than it eats
	Ladybird beetle (*Coccinella septempunctata*)	500–1000 adults	p.r.n. every 3 wk	Average efficacy, very inexpensive—cost efficient
	Green lacewing (*Chrysoperla rufilabris*)	1000–5000 eggs	Monthly (mo), low‐quantity bottles as needed for outbreaks	Effective slow release from cards, mediocre success from loose release
	*Aphidius* wasp (*Aphidius colemani*)	500 mummies	4–6 wk before expected aphids	Works well in parallel with *C. septempunctata*
	*Aphidius* wasp (*Aphidius matricariae*)	250 mummies	Bimonthly (2 mo), p.r.n.	—
Ant (Formicidae)	—	—	—	Used as an indicator for aphid detection
Fungi (Erysiphales)	Preventive measures, spray treatment	—	p.r.n.	Preventive measures greatly reduced outbreaks
Fungus gnat (Mycetophilidae)	Scanmask (*Steinernema feltiae*)	1 L per 1000 sq ft	p.r.n.	Near immediate results, consumes all pest and beneficial soil‐dwelling insects
	*Hypoaspis miles*	1 L per 1000 sq ft	p.r.n.	Immediate results
Glasshouse thrips (Thripidae)	Predatory mite (*Amblyseius* [*=Neoseiulus*] *cucumeris*)	100 mini sachets	Bimonthly (2–3 mo)	Slow‐acting sachets (5–7 wk), effective preventive measure
	Minute pirate bug (*Orius insidiosus*)	500 nymphs to adults	p.r.n.	Highly effective as a predator for outbreaks, population longevity without prey or replenishment
Glasshouse whitefly (*Trialeurodes vaporariorum*)	Predatory wasp (*Encarsia formosa*)	1000 (10 100‐count egg mini sachets)	Monthly (mo)	No outbreaks, highly effective as preventive measure
Mealybug (Pseudococcidae)	Ladybird beetle (*Cryptolaemus montrouzieri*)	100–1000 adults	Weekly (wk), large quantity for outbreaks	Highly effective if released directly on infestation, immediate results
Two‐spotted spider mite (*Tetranychus urticae*)	Predatory mite (*Phytoseiulus persimilis*)	2000 eggs to larvae	Monthly (mo)	Effective for outbreaks, inexpensive, dies quickly without prey
	Predatory mite (*Mesoseiulus longipes*)	1000 mites	Weekly (wk) p.r.n.	Thrives in high‐temperature greenhouses
	Predatory mite (*Neoseiulus californicus*)	1000 mites	Weekly (wk) p.r.n.	Slower acting, survive for longer periods without prey
Flea beetle (*Epitrix cucumeris*)	Scanmask (*Steinernema feltiae*)	1 L per 1000 sq ft	p.r.n. biweekly (2 wk)	High efficacy with repeated use, consumes other soil‐dwelling insects

Note

p.r.n. = as needed.

*Aphidoletes aphidimyza* (Rondani), the aphid gall midge, were released as adults and served as an effective, quick‐acting beneficial insect for addressing aphid outbreaks (Tables 1, 2). The midges were noted for their efficacy in dealing with aphid infestations, killing more aphids than they actually consumed. Midge larvae established in aphid colonies produce rapid eradication of adult aphids.

Two ladybird beetles, *Coccinella septempunctata* L. and *Hippodamia convergens* Guérin‐Menéville (Fig. 1C, D), have been used in conjunction with both gall midges and lacewings as another cost‐effective, quick‐acting beneficial insect (Tables 1, 2). Released as adults, ladybird beetles are highly effective as a long‐term, multi‐generation approach to aphid control. Adults have been observed preying on aphid populations within minutes of release. Ladybird beetles populations are often maintained at low levels through sustained sexual reproduction, as evidenced by the presence of larvae on study plants. Orders of ladybird beetles every 6–10 weeks are enough to sustain large, active populations during aphid outbreak seasons in our greenhouses.

Aphid parasitic wasps, *Aphidius colemani* Viereck and *A. matricariae* Haliday, are relatively effective for the prevention and management of small aphid populations (Tables 1, 2). *Aphidius colemani* was more commonly used, with *A. matricariae* being used only once in an extreme outbreak. Released as emerging adults, both wasp species could immediately begin laying eggs, beginning the endoparasitism complex. Both wasp species were significantly less effective than other beneficial insect controls for aphids due to their longer life cycles, lack of multi‐prey consumption, and cost. Because they require a nectar resource, we grow sweet alyssum (*Lobularia maritima* (L.) Desv.) alongside research plants. The wasps are most effective when substantial levels of aphids are available, although *A. colemani* was noted for its ability to remain present in the absence of aphid outbreaks and then rising again with seasonal emergence of aphids. Parasitized aphids take time to mature while they are still alive, and must not be disturbed while the eggs of the wasp are developing. Although this provides opportunities for students to observe the life cycle of the parasite, it also might test the patience of those involved in reducing the aphid population.

#### Ants (Formicidae)

Ants are occasionally observed sparsely distributed throughout the greenhouse. Common store brand ant traps were viewed as acceptable methods of countering swelling ant colonies if needed, and have posed little to no threat to plant and other beneficial insect life. In other environments, ants may pose a greater threat to predator–prey equilibrium.

#### Powdery mildew (Erysiphales)

Powdery mildew was commonly observed on plants in early stages of greenhouse IPM establishment (Table 2). The high temperatures of the greenhouses along with the humidity caused by regular watering led to the rapid growth and spread of such infections. To counter fungal establishment, low‐spray aluminum water breakers (Dramm Corporation, Manitowoc, Wisconsin, USA) were used, limiting the amount of water sprayed into the air when watering at soil level. Plants were watered during morning hours and leaves were elevated out of direct contact with water and damp soil, aiding in aeration. Basal leaves infected with fungal growth were removed when noted. The use of these techniques effectively limited establishment of new infections. Severe infections are treated with a mixture of baking soda, vegetable oil, natural dish soap, and water sprayed directly on leaves in the afternoon when cool or overcast. (Mixture: for every 2 L water, add 2 teaspoons baking soda, 1–2 mL of vegetable oil, and 1 small squirt of soap.)

#### Fungus gnats (Mycetophilidae)

Outbreaks of Mycetophilidae species (fungus gnats) were universally damaging across all species at early vegetative growth stages, stunting plant growth for short periods of time. Fungus gnat larvae feed on new root growth, and preventive measures aimed at limiting gnat population growth initially yielded little success. Biological controls through the use of *Hypoaspis miles* Berlese, soil‐dwelling mites, and Scanmask (BioLogic Company) soil nematodes (*Steinernema feltiae* (Filipjev) [Two close parentheses is correct here. Please make the change as marked previously.]) were applied separately with high efficacy for both controls (Tables 1, 2). The cycling of Mycetophilidae controls around other beneficial insect life cycles is critical to successful IPM maintenance as soil nematodes and mites may be harmful to soil‐dwelling stages of other beneficial insects (e.g., *A. aphidimyza*). Other growers have successfully employed the organic larvicide Gnatrol (Valent, Walnut Creek, California, USA; Theresa Culley, University of Cincinnati, personal communication). Whatever is prescribed, we have found that overwatering is the key trigger for fungus gnat infestations; close attention to this issue has greatly reduced the need for treatments.

#### Thrips (Thripidae)

Through the use of the predatory mite *Amblyseius cucumeris* (Oudemans) as a preventive measure, large thrip outbreaks never occurred in our greenhouse, although the pests were repeatedly present in low numbers (Tables 1, 2). *Amblyseius cucumeris* proved to be a highly economical control, used regularly from slow‐release sachets and introduced into the soil via shaker tubes during transplanting of plants. Establishment of *A. cucumeris* populations in the soil during repotting and continued renewal via slow‐release sachets was vital to the mites’ efficacy in preventing thrip outbreaks by feeding on immature, soil‐dwelling stages. When adult thrips were present, *Orius insidiosus* (Say), minute pirate bugs, were released as adults as an immediate, quick‐acting biological control (Tables 1, 2). *Amblyseius cucumeris* and *O. insidiosus* were suited as cost‐effective, long‐term thrip control options, with adults being able to feed off pollen in the absence of thrips. *Orius insidiosus* was particularly well adapted for greenhouse life, with an active population self‐maintaining without additional releases. Population levels of *O. insidiosus* were relatively effortless to track as adults and young stages are often found dwelling in between the stamens of flowers during daylight hours. The sustained, although limited, pollen consumption by *O. insidiosus* in the absence of thrips has had no measurable adverse effect on ongoing research projects.

#### Whitefly (*Trialeurodes vaporariorum* Westwood)

The common parasitic wasp, *Encarsia formosa* Gahan, is used with high efficacy as a slow‐release (hanging card) preventive control against whitefly (Tables 1, 2). Introduced to coincide with the beginning of our IPM program, *E. formosa* is recommended for preventive release during low cycles of whitefly populations due to their ability to prey on immature whitefly stages. Adults wasps were observed during dusk hours on leaf surfaces and the emergence holes of young adults were clearly visible on parasitized whitefly pupae located on leaf undersides.

#### Mealybugs (Pseudococcidae)

Mealybugs are detrimental to *Solanum* plants, causing damage by sucking plant sap from leaf veins, resulting in rapid defoliation and stunted or terminated growth. This common pest is one of the easiest of all pests to scout, with expanding populations usually found in branch nodes, under the calyces of flowers, and in between developing flower buds within inflorescences. *Cryptolaemus montrouzieri* Mulsant (Fig. 1E, F), commonly known as “crypts” and “mealybug destroyers,” is a species of ladybird beetle we regularly use in the greenhouse for control of mealybug populations (Table 1). Highly effective in reducing outbreaks, crypt adults would begin preying on adult mealybugs within several minutes of release (Tables 1, 2). Due to their large size and accessibility, mealybugs can also be hand squashed or removed with a small brush (such as a toothbrush) when infestations occur. However, the similarity of appearance between crypt larvae and adult mealybugs can make successful hand‐squashing attempts difficult and counterproductive to untrained eyes, an important distinction to transfer to untrained and new students. Continued release of adult crypts has successfully eliminated mealybug populations and prevented resurgence in our greenhouse.

#### Spider mites (*Tetranychus urticae* Koch)

Predatory mites of *Phytoseiulus persimilis* Athias‐Henriot were introduced to the greenhouse in egg sachets from which predators slowly hatched over time (Tables 1, 2). This species feeds on all stages of spider mites and is inexpensive for active control when released as adults, but will need continual release to maintain an active population. These predatory mites were effective as an active and preventive measure, but once spider mite outbreaks become established, *P. persimilis* is less effective at suppressing additional generations without continual supply. When outbreaks do occur, two additional predatory mites, *Mesoseiulus longipes* (Evans) and *Neoseiulus californicus* (McGregor), are released in their adult stages, directly targeting all stages of spider mites (eggs through adults). *Mesoseiulus longipes* was brought in for its efficacy in high‐temperature greenhouses, whereas *N. californicus* is known for surviving even in the absence of spider mite populations. All three predatory mites were effective for several generations of *Solanum* growth, but without continual release spider mite populations grew out of control and could not be suppressed without chemical (neem oil and/or mild chemical sprays) treatment, especially during summer months when some predatory species may be more sensitive to higher temperatures than others. It should be noted that *P. persimilis* eggs are sometimes eaten by the thrips predator *A. cucumeris*, so release of both beneficials should be carefully timed or quantities adjusted.

#### Flea beetles (*Epitrix cucumeris* (Harris))

Low‐level populations of flea beetles left unattended can quickly develop into difficult‐to‐control outbreaks. With a larger body than most pests (1.5–3.2 mm) and obvious damage (large holes) left in leaves and flower petals from feeding, flea beetles are easy to scout for and detect. The large size and high mobility of adult flea beetles effectively combats conventional physical and chemical controls on adult plants. Mechanical hand squashing of adult stages can be used to aid in suppression of larger outbreaks. Scanmask (*S. feltiae*) was used with great success to control *E. cucumeris* populations by targeting the soil‐dwelling egg, larval, and pupal life stages (Tables 1, 2). Although highly useful in treating fungus gnats and flea beetles, soil nematodes will consume other soil‐dwelling insects and their use should be scheduled to accommodate subterranean life stages of other beneficials. During outbreaks, Scanmask was applied to soil, benches, and floors as recommended every two weeks until individuals of *E. cucumeris* were no longer found. For long‐term maintenance, Scanmask can be used on a 4–6‐month schedule for overall soil‐dwelling pest problems.

### Chemical control

Chemical control has only been used as a last‐resort treatment for pest infestations. Mild chemical sprays are used to treat infestations when plant mortality increases due to pest exposure or when recalcitrant pests are otherwise unable to be eradicated. Neem (*Azadirachta indica* A. Juss.) oil spray is used to spot treat mealybug populations and has at times been extensively employed in severe cases of spider mite infestation. Additional mild products (e.g., Safer soap spray; Woodstream Corporation, Lititz, Pennsylvania, USA) are purchased as needed, although there is a plethora of such products available for retail purchase. Natural homemade mild pesticides could also be employed to limit spending. With plenty of recipes available through a quick internet search, this is a cheaper alternative, but, as with any chemical control, should be tested before applying in bulk. We choose to use chemical controls sparingly and only when necessary for treating large initial outbreaks. Because beneficial insects arrive every two weeks, chemical controls are timed to the alternating weeks, as much as possible.

### Thoughts on IPM for combined research and teaching collections

Due to the nature of our study system, our pest‐specific thresholds varied very little as the loss of one plant due to pest pressure could set projects back by months or years. Likewise, the health of flowers is key to our research on reproductive biology. Chemical controls can serve as a hard stop to prevent pest infestation and flower, fruit, or plant loss. It is important to outline the necessities of ongoing research projects in deciding when to employ chemical controls as a last‐resort measure. Establishing an IPM schedule for each research project will help ensure success. In the case of taxonomic research collections and teaching collections, other aspects may be more important, such as root health for long‐term culture. At Bucknell University, our rooftop greenhouse shares a wall with (but is not connected to) the permanent teaching collection greenhouse that has been using IPM for almost 20 years. The IPM schedule was established in the same way as our research program schedule, and is maintained by the greenhouse manager, Wanda Boop. The overall goal of using IPM is not to eliminate chemicals as a control, but to reduce the use of them. Use of IPM will also reduce the resistance of pests to certain chemicals which ultimately results in the need to use chemicals of increasing toxicity. Because students participate in greenhouse care and/or research at most academic settings, reducing their exposure to toxic chemicals, both during application and through residues left behind, is a persistent goal for implementing IPM in academic settings.

## DISCUSSION

Over the past several years, we have successfully established a biological control–focused IPM program that accounts for novel pest outbreaks, seasonal variation in pest populations, and rapid unpredictable outbreaks. By establishing cultural, mechanical, and physical controls prior to plant culture, we have largely supplanted chemical controls with biological controls in our growing spaces (except for rare events).

A reasonable question is: What does this all cost? Committing to an IPM program does require persistent funding if continual culture of research plants is required, but it is largely free of “big ticket” purchases that might be associated with other pest/pathogen management strategies. To maintain populations of beneficial insects in our two small research greenhouses at Bucknell, we are currently receiving an IPM shipment every two weeks costing between US$90–$105 per order, on average. The estimated annual cost to cover those scheduled shipments plus additional supplemental orders made during seasonal outbreaks is about US$2500 per year (roughly US$1250 per greenhouse).

Our investment in a sustainable pest management strategy has been integral to establishing and maintaining a successful scholarly trajectory for the botany lab at our primarily undergraduate institution. Particularly, the ability to maintain healthy ex situ populations of study plants has expanded our options for research and allowed for further development of projects requiring extended life history observations such as reproductive biology studies (e.g., Martine et al., 2016a), morphometric comparisons (e.g., Martine et al., 2016b; McDonnell et al., 2019), and description of new taxa for which herbarium material was limiting (e.g., Lacey et al., 2017). Our sustained living collections also act as repositories of fresh plant material for use in studies employing molecular data sets (e.g., Martine et al., 2019). These benefits have come without the costs of pesticides and the associated potential health risks for student researchers. At the same time, the commitment to IPM has meant that some types of research questions cannot be addressed in our facilities without modifications to our plan. For example, the constant low‐level presence of insects correlates to base levels of plant defense chemicals that may confound experiments related to plant–herbivore responses (Rupesh Kariyat, University of Texas, Rio Grande Valley, personal communication). Likewise, even small numbers of floral pests (e.g., thrips) or their predators might be enough to dissuade bees from visiting flowers during studies on foraging behavior (Avery Russell, Missouri State University, personal communication).

## AUTHOR CONTRIBUTIONS

D.S.H., I.E.J.T., and C.T.M. established the initial IPM strategy; I.E.J.T., J.T.C., and A.J.M. managed the IPM systems; D.S.H. and C.T.M. led the manuscript writing.

## Overview of common nutrient deficiencies encountered during *Solanum* spp. growth. Healthy plants are less susceptible to pests and thus require less IPM investment.

**Table:**

Nutrient	Deficiency symptoms	Beneficial treatment	Comments	References
Macronutrients				
Nitrogen (N)	Older leaves will display signs of spotty to general yellowing; rest of the plant will fade to lighter green	Foliar spray: 21%; osmocote: 15%; perlite .07%	As needed or every 2 mo; beneficial during crossing experiments	Zhao et al. (2005); *Solanum*: Urbanczyk‐Wochniak and Fernie (2005)
Phosphorus (P)	Underside of leaves will turn dark green to purple, tips will appear burnt	Foliar spray: 18%; osmocote: 9%; perlite .07%	As needed or every 3 mo	Stewart et al. (2001); *Solanum*: Baylis (1970)
Potassium (K)	Older leaves appear scorched, wilted; interveinal chlorosis begins from leaf margins	Foliar spray: 21%; osmocote: 12%; perlite .07%	As needed or every 2 mo	Coleman and Richards (1956); *Solanum*: Pettigrew (1999)
Micronutrients				
Copper (Cu)	Stunted plant growth; leaves turn dark green	Foliar spray: 0.05% osmocote: 0.05%	Covered by NPK fertilization	Arnon and Stout (1939), Yruela (2005)
Iron (Fe)	Yellowing of veins in young leaves	Foliar spray: 0.10%; osmocote: 0.05%	Covered by foliar sprays during fruit set	Clark (1982), Lucena (2006); *Solanum*: Chatterjee et al. (2006)
Zinc (Zn)	Yellowing of veins in young leaves; rosetting of terminal leaves	Foliar spray: 0.05%; osmocote: 0.05%	Covered by NPK fertilization	Cakmak and Marschner (1987), Randall and Bouma (1973)

Note

NPK = nitrogen, phosphorus, potassium.
